# Increasing awareness of food-choking and nutrition in children through education of caregivers: the CHOP community intervention trial study protocol

**DOI:** 10.1186/s12889-019-7469-7

**Published:** 2019-08-22

**Authors:** Giulia Lorenzoni, Danila Azzolina, Solidea Baldas, Gianni Messi, Corrado Lanera, Megan A. French, Liviana Da Dalt, Dario Gregori

**Affiliations:** 1 Unit of Biostatistics, Epidemiology and Public Health, Department of Cardiac, Thoracic, Vascular Sciences and Public Health, University of Padova, Via Loredan, 18, 35131 Padova, Italy; 2 Department of Translational Medicine, University of Piemonte Orientale, Novara, Italy; 3 Prochild Onlus, Trieste, Italy; 4 Department of Pediatrics, Burlo Garofolo Hospital, Trieste, Italy; 5 Division of Pediatric Emergency Medicine, Department of Women’s and Children’s Health, University of Padova, Padova, Italy

**Keywords:** Choking prevention, Foreign body, Public health, CHOP community intervention trial

## Abstract

**Background:**

Choking is one of the leading causes of death among unintentional injuries in young children. Food choking represents a considerable public health burden, which might be reduced through increased effective preventative education programs. We present a protocol for a community intervention trial termed CHOP (CHOking Prevention project) that aimed to teach Italian families how to prevent food choking injuries and increase knowledge relating to nutrition.

**Methods:**

Italian educational facilities were enrolled. Stratified randomization blocked by geographical area was performed. Each stratum was randomized to one of three different intervention strategies or to a control group. Educational intervention was delivered in the schools by experts and certified trainers as per the following three intervention strategies: directly to families (Strategy A); to teaching staff only, who subsequently delivered the same educational intervention to families (Strategy B); to health service staff only, who then delivered the educational intervention to teaching staff, who subsequently delivered the intervention to families (Strategy C).

Participants completed a questionnaire about their knowledge on the topics presented during the educational interventions (pre-, post-, and follow-up of intervention). Information from the questionnaires was synthetized into 6 indicators in order to measure how effective each intervention strategy was.

**Discussion:**

The issue of food choking injuries in children is relevant to public health. The protocol we present provides an opportunity to progress towards overcoming such challenges through a working model that can be implemented also in other countries.

**Trial registration:**

ClinicalTrials.gov NCT03218618. The study was registered on 14 July 2017.

## Background

Historically, injuries were considered “accidents” occurring by chance. However, increased knowledge about factors that affect the occurrence of such events has raised awareness about the fact that they may be predictable and preventable, and that they can therefore be referred to as “injuries” as oppose to “accidents” [1, 2]. This also applies to choking injuries. In recent years, the development of prevention strategies for non-food choking injuries has resulted in a reduction of the occurrence of this type of injury. For example, injuries due to toys (and toy parts) decreased after the development and implementation of an ad-hoc regulation on toy safety and associated public health campaigns [3], which informed adult supervisors on the importance of choosing certified toys and nursery products.

Insufficient attention has, however, been given to food related choking injuries even though some studies have shown that caregivers often lack knowledge about this issue. For example, Nichols et al. [4] conducted a survey among parents of infants and toddlers relating to food and non-food hazardous items, and about the actions considered necessary to prevent choking injuries. Interestingly, knowledge about hazardous non-food items was found to be higher than knowledge of food items, and parents who were unaware of food related choking hazards were more likely to give their children foods that pose a high risk of choking. Consistent with these findings are those of a survey conducted on Japanese mothers with children under the age of two [5], which showed that the mothers did not consider nuts and seeds (which are the food items most frequently involved in choking episodes [6]) as hazardous food items to be avoided by infants and toddlers. Additionally, Susy Safe data, perhaps one of the world’s largest registries collecting data on foreign body (FB) injuries in children aged 0–14 years old [7], shows that about 40% of recorded food injuries occurred while children were eating without adult supervision. The remaining 60% occurred when children were eating improper food (or food prepared improperly) despite adult supervision [8]. These findings denote a concerning lack of knowledge about choking hazard among caregivers.

The need for choking prevention strategies is evidenced by epidemiological data [9]. Choking is one of the leading causes of death among unintentional injuries in young children, and remains relevant until the age of 14 [10]. Additionally, food-related choking injuries account for up to 60–80% of all choking injuries in some studies [11]. This is due in part to physiological characteristics of children that make them more prone to choking while eating [12]. Food items most frequently involved in FB injuries share specific characteristics of size, consistency/texture, and shape, which make them difficult to chew for young children [13]. Consequently, food items may be swallowed before the child has chewed them sufficiently. Depending on the size and texture of the item involved, once in the airways they can conform to the pharynx and cause a blockage or reach the lower respiratory tract.

Despite the huge burden on public health services that is consequence of food choking injuries, public health programs in this field are sparse, and only a few studies have evaluated the effectiveness of such strategies. However, two studies that were conducted in Israel [14] and in Crete [15] showed that a reduction of choking cases in children occurred after the implementation of educational programs on choking prevention.

Here we report on the CHOP (CHOking Prevention) study protocol, which evaluated the effectiveness of public health intervention on food choking by comparing three different school-based intervention strategies. The CHOP project aimed to: (i) teach families how to prevent food choking injuries in children; (ii) inform policy-makers on the effectiveness of community-based intervention schemes as oppose to intervention on an individual basis; (iii) determine the intervention strategy that offers the best compromise between educational effectiveness and cost effectiveness. The expectation is that the optimal strategy will be introduced into schools across Italy as the first Italian public health intervention program to combat the issue of food choking.

## Methods/design

### Trial design

The CHOP project was designed as a community intervention trial to compare three different school-based intervention strategies. The project delivered education on primary and secondary food choking prevention and nutrition under the umbrella topic of nutrition education, and was designed with the intension of reaching as many people as possible through the participation of families in schools. The study protocol followed the SPIRIT statement [16].

### Study setting

Italian educational facilities (nurseries, kindergartens, and primary schools) were included in the trial. Educational facilities were stratified such that each stratum included an educational facility from each educational stage (i.e., each stratum includes one nursery, one kindergarten, and one primary school). Healthcare staff, teachers, and families (parents or guardians) of children attending the schools participated in the project. The project did not involve children (under 16 years of age) but only families (parents or guardians).

Study particpants (healthcare staff, teachers, and families -parents or guardians-) were informed about the nature and the scope of the study by study coordinators. If they agree to participate, they must provide written informed consent in accordance with Ethical Committee (EC), using an EC approved informed consent form, including the consent *for the use and processing of their personal details.*

### Eligibility criteria

Eligibility criteria for school participation was as follows:

- availability of at least one subject from the school’s health service;
- availability of a room with suitable technology (monitor, sound system) for educational intervention;
- approval of the school’s inclusion by the competent authority.

Also, parents or guardians, school health service personnel, and teaching staff had to be:

- aged between 18 and 60 years old;
- without hearing or eyesight impairments;
- consenting to the project participation.

### Interventions

**Fig. 1:**
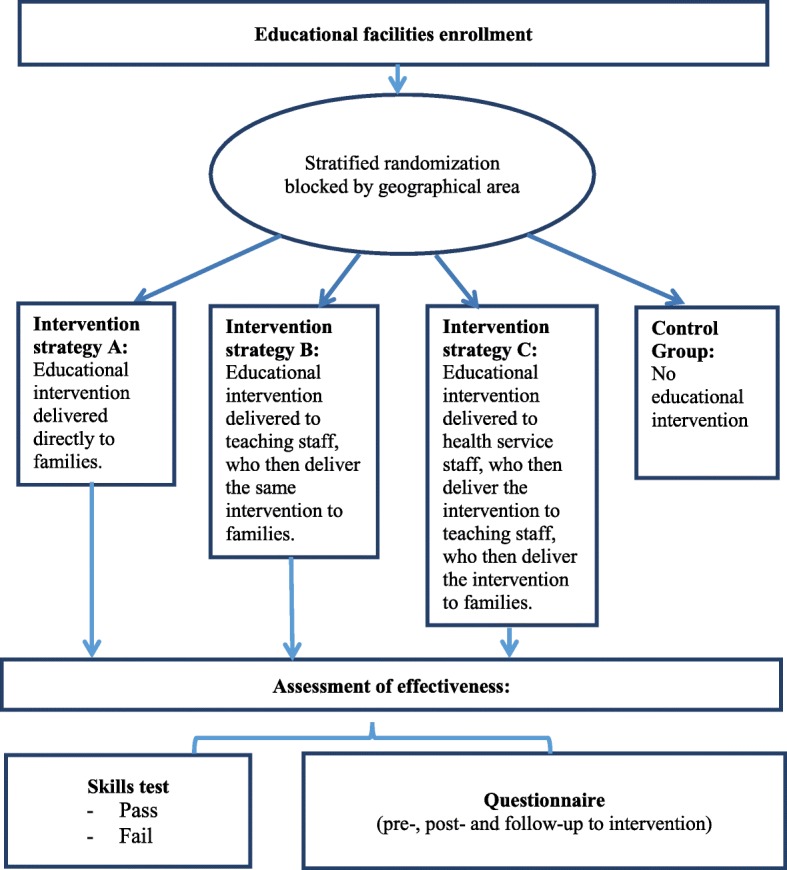
Flow-chart of the CHOP study

Educational intervention was delivered in the schools by experts and certified trainers as per the following three intervention strategies (Fig. 1).

- Strategy A: directly to families.
- Strategy B: to teaching staff only, who subsequently delivered the same educational intervention to families.
- Strategy C: to health service staff only, who then delivered the educational intervention to teaching staff, who subsequently delivered the intervention to families.
- Control group: no educational intervention.

The same educational intervention was delivered in all strategies and consisted of the following:

- A lecture on primary prevention of food choking and on nutrition (see below) given by experienced trainers.
- Training on maneuvers to dislodge FBs (secondary prevention) demonstrated by trainers certified by the Italian Society of Pediatric Emergency Medicine (SIMEUP).
- Distance education via a Massive Open Online Course (MOOC) to reinforce the lecture content (see below).

Lecture content included:

- Primary prevention of food choking injuries (epidemiology of the phenomenon; characteristics that make children more prone to choke than adults; obstruction mechanisms; detailed description of hazardous food products; information on the characteristics of FBs (shape, texture, and size) that are associated with a high risk of aspiration; recommendations for food preparation).
- Basic information regarding food labels, including an explanation of the types of information reported on labels and their meaning).
- Tips for food waste prevention.

The lecture and the training were carried out on the same day. At the end of the session, participants were asked to subscribe and join the MOOC to reinforce their learning. The MOOC is an innovative learning modality and consists of a series of free and brief videos that are available on a dedicated website (www.safefood4children.org). The online course is available in English, Italian, and Italian sign language, and will eventually be available in French, Arabic, Chinese, Japanese, Malay, and Portuguese. In each MOOC video, a field expert (a professor of biostatistics and epidemiology, an ENT doctor, a pediatrician, a researcher, and a professional chef de cuisine) presents on the following topics:

- epidemiology of choking injuries;
- psycho-physiological characteristics that make children more prone to choke;
- FB obstruction mechanisms;
- hazardous food items;
- food preparation;
- hazardous non-food items;
- first aid and maneuvers for pediatric unblocking;
- food labeling;
- food waste prevention.

### Assignment of interventions

Stratified randomization blocked by geographical area was performed. Each stratum was randomized to one of three different intervention strategies or to the control group (Fig. 1). The randomization was blocked by geographical area (Northern, Central, Southern, and major Islands). Participants and trainers were not blinded to strata allocation, but those in charge of data analyses and interpretation of results were blinded.

### Data collection methods

Data collection differed for participants involved in the intervention strategies (A, B, C) and in the control group -since they did not undergo the educational intervention-, even though the instruments employed were the same.

Before the educational intervention, participants in each intervention strategy (A, B, C) completed a socio-demographic questionnaire and a questionnaire about their baseline knowledge on food choking prevention, food labeling, and food waste prevention. A questionnaire was then completed by participants involved in each intervention strategy (A, B, C) to obtain data relating to taught material, with questionnaire being completed, i) immediately after (post), and ii) 1 month after (telephone follow-up) educational interventions. A skills test was also given to them immediately after the educational intervention and consisted of a checklist to evaluate their ability to perform maneuvers to dislodge FBs and thus assess the effectiveness of the secondary prevention training.

Participants involved in the control group completed only once a telephone-administered socio-demographic questionnaire and a questionnaire to assess their knowledge on food choking prevention, food labeling, and food waste prevention (the same questionnaire was administered to participants involved in each intervention strategy immediately after and 1-month after the educational intervention).

### Outcomes

Participants were assessed as having either passed or failed the skills test. The responses from the questionnaire (completed at post-, and follow-up stages) were synthesized into indicators in order to measure how effective each intervention strategy (A, B and C) was in improving knowledge about primary prevention of food choking. Four indicators (1–4, Table 1) based on different topics relating to choking, and two indicators (Table 2) for nutrition topics, were calculated for each stage of each strategy. Weighting of the answers within each indicator is shown in Table 1 for choking education and Table 2 for the nutrition education.

**Table 1:** Indicator topics, questions, and weighted scores used to assess CHOP choking educational intervention strategies

Indicator	Topic	Question	Weight of question
1	Epidemiological knowledge	Do you know why children are at risk of choking?	0.33
		At what age are children at highest risk of choking?	0.33
		How many deaths per year are estimated to result from foreign body injuries in EU countries in children between 0 and 14 years of age?	0.33
2	Risk Perception	Are magnets, if swallowed in numbers greater than one, dangerous?	0.15
		What objects are most frequently involved in foreign body injuries?	0.35
		What objects cause the most serious and fatal injuries?	0.35
		Why are button batteries dangerous if ingested?	0.15
3	Rules for food preparation	When should it be assumed that a child has inhaled a foreign body, and what should be done?	0.1
		What size should food be prepared to?	0.1
		How should we prepare and cook meat and fish to reduce the risk of choking and injury?	0.3
		How should you cut wurstel and hot dogs?	0.3
		What should children do during meals and when eating?	0.1
		Do particular food preparation techniques help to reduce the risk of choking?	0.1
4	Ability to recognize hazardous foods	Which food represents a high risk of choking to children?	0.4
		Why is food of a round shape hazardous?	0.4
		Why do we have to give babies nuts in a ground form incorporated to other foods with a soft consistency (e.g., yogurt)?	0.1
		At what age can children be given candies and sweets?	0.1

**Table 2:** Indicator topics, questions, and weighted scores used to assess CHOP nutrition educational intervention strategies

Indicator	Topic	Question	Weighted score
1	Knowledge about nutritional indications and food labeling.	Identification of the obligations for producers with regard to food labeling introduced by regulation n. 1169/2011.	0.5
		Definition of nutritional indication reported by regulation n. 1924/2006.	0.5
2	Knowledge about the prevention of food waste.	Identification of the precautions to reduce food waste at home.	0.5
		Definition of the minimum terms of conserving food.	0.5

### Sample size

Sample size estimation was performed, as recommended for such types of studies [17], by taking into account the intra-cluster correlation coefficient (ICC), *ρ*, and the intra-stratum correlation coefficient, *ρ*_*m*_ (where *m* is the number of strata). Equation 1 shows the recommended approach for calculating the difference in mean outcome considering multiple comparisons for continuous outcomes [18].

1 $$ {n}_i=\frac{2{\left({z}_{\frac{\alpha }{2 nc}}+{z}_{\beta}\right)}^2{\sigma}^2}{{\left({\mu}_1-{\mu}_2\right)}^2} $$

Where *n*_*i*_ is the estimated sample size for *i* = 1, 2 groups, *nc* is the number of pairwise comparisons, and *μ*_1_ − *μ*_2_ is the expected difference in proportions among groups, *α* is the significance level and *β* is the probability of Type II error in any hypothesis test.

In this research framework, it is important to consider a sample size estimation procedure that takes the ICC, *ρ*, and intra-stratum, *ρ*_*m*_, into account. An approach that does this is provided by Crespi [17] and is represented by Eq. 2. Following this approach, the overall sample size is:

2 $$ \frac{4{\left({z}_{\frac{\alpha }{2 nc}}+{z}_{\beta}\right)}^2{\sigma}^2\left[1+\left(m-1\right)\rho -m{\rho}_m\rho \right]}{{\left({\mu}_1-{\mu}_2\right)}^2} $$

The sample size was calculated for three pairwise comparisons between the intervention and control groups [19] for the difference in mean outcome, and with consideration of a Bonferroni correction on the *α* significance value [18]. The sample size was calculated according to the following assumptions:

- *α* = 0.05 (adjusted for three pairwise comparisons).
- Power = 0.8
- *m* = 4 (minimum number of families in schools).
- *nc=3* pairwise comparisons
- We assume *ρ* = 0.05. This is based on a study that reported the same expected value though undertaking a Cluster Randomized Controlled trial for evaluating an injury prevention education program in 20 primary schools [20].
- Intra stratum correlation, *ρ*_*m*_ = 0.05 (as assumed for *ρ*)
- A difference in mean of the correct answer corresponding to an effect of 0.183.
- A pooled variance *σ*^2^ for the difference in means outcomes equal to 1
- A baseline mean *μ*_1_ of the correct answer as 0.7.

The resultant sample size that we determined in the trial was 1426.

The study size was adjusted taking into account a 15% drop out rate; for this reason 1670 participants in 48 schools (35 per-school) were enrolled.

Computations were performed using the ‘R’ System (Version 3.3.2) [21].

*Statistical methods.*

Indicators were reported as mean (± standard deviation). Wilcoxon-Kruskal-Wallis tests were computed in order to compare each intervention strategy with the control group at each study stage (post and follow-up): i) Strategy A vs. Control Group ii) Startegy B vs. Control Group iii) Strategy C vs. Control Group. The term of comparison (Control Group) was the same at both study stages (post and follow-up) since participants involved in the control group underwent questionannire administration only once. To account for multiplicity of testing, *p*-values were adjusted according to the *Benjamini-Hochberg* procedure at each study stage (post and follow-up). In addition to that, to account for potential confounders (socio-demographic characteristics and baseline knowledge on choking prevention, food labeling, and food waste prevention), regression models were estimated, one for each indicator, at both study stages (post and follow-up).

## Discussion

The issue of food choking injuries in children is relevant from a public health perspective, but it is often neglected [22]. Italian public health interventions are scant, and only a few initiatives have been made globally to reduce the public health burden of food choking injuries. The United States and Sweden are the only countries to have developed specific regulations aimed at choking prevention [23–25]. In Italy, there are no other known initiatives to address and combat this concerning public health issue. Training on food choking prevention is currently done on a voluntary basis with families incurring costs themselves. The cost of training can represent a considerable economic burden for low-income Italian families, and this creates inequality in the ability to access to choking prevention training. This is especially concerning given that children from families with a low socio-economic status (SES) are more prone to choking, and more likely to experience injuries in general, in comparison to families with a medium-high SES [26].

The timing of the delivery of educational intervention on food choking is critical if families are to be trained before solid foods are introduced into their children’s diets (usually at around 6 months of age). Ideally, some training should be provided before children are born (e.g., during prenatal classes). However, in Italy, a school-based intervention currently represents the most feasible and realistic way to reach as many families as possible because school attendance is compulsory, whereas other occasions for parents-to-be and existing parents to meet are not compulsory (e.g., prenatal class). It has also been demonstrated, for example, that non-compulsory classes are inadequately attended by migrants [27], thus producing further inequalities in the ability to access food choking prevention training.

The protocol we present, based on the CHOP community intervention trial, provides an opportunity to overcome such challenges through a working model that can be implemented in other countries in order to reduce the public health burden of food choking injuries in children and increase nutritional education to caregivers.

## Data Availability

The datasets used and/or analysed during the current study are available from the corresponding author on reasonable request.

## Abbreviations

CHOP: CHOking Prevention project
FB: Foreign Body
MOOC: Massive Open Online Course
SES: Socio-Economic Status
